# Chronic Monocular Deprivation Reveals MMP9-Dependent and -Independent Aspects of Murine Visual System Plasticity

**DOI:** 10.3390/ijms23052438

**Published:** 2022-02-23

**Authors:** Sachiko Murase, Sarah E. Robertson, Crystal L. Lantz, Ji Liu, Daniel E. Winkowski, Elizabeth M. Quinlan

**Affiliations:** 1 Department of Biology and Neuroscience and Cognitive Sciences Program, University of Maryland, College Park, MD 20740, USA; crystal.lantz@nih.gov (C.L.L.); liuji1031@gmail.com (J.L.); danwinkowski@gmail.com (D.E.W.); equinlan@umd.edu (E.M.Q.); 2 Department of Bioengineering and Biomedical Engineering, University of Maryland, College Park, MD 20740, USA; srobert3@terpmail.umd.edu

**Keywords:** chronic monocular deprivation, MMP9, ocular dominance, calcium imaging

## Abstract

The deletion of matrix metalloproteinase MMP9 is combined here with chronic monocular deprivation (cMD) to identify the contributions of this proteinase to plasticity in the visual system. Calcium imaging of supragranular neurons of the binocular region of primary visual cortex (V1b) of wild-type mice revealed that cMD initiated at eye opening significantly decreased the strength of deprived-eye visual responses to all stimulus contrasts and spatial frequencies. cMD did not change the selectivity of V1b neurons for the spatial frequency, but orientation selectivity was higher in low spatial frequency-tuned neurons, and orientation and direction selectivity were lower in high spatial frequency-tuned neurons. Constitutive deletion of MMP9 did not impact the stimulus selectivity of V1b neurons, including ocular preference and tuning for spatial frequency, orientation, and direction. However, MMP9^−/−^ mice were completely insensitive to plasticity engaged by cMD, such that the strength of the visual responses evoked by deprived-eye stimulation was maintained across all stimulus contrasts, orientations, directions, and spatial frequencies. Other forms of experience-dependent plasticity, including stimulus selective response potentiation, were normal in MMP9^−/−^ mice. Thus, MMP9 activity is dispensable for many forms of activity-dependent plasticity in the mouse visual system, but is obligatory for the plasticity engaged by cMD.

## 1. Introduction

The shift in ocular preference in binocular neurons of the primary visual cortex is the canonical model of receptive field plasticity confined to a postnatal critical period. In mice, the critical period for ocular dominance plasticity peaks during the third postnatal week, after one week of binocular vision. During the critical period, MD induces an early decrease in excitatory drive onto parvalbumin-expressing interneurons, which is permissive for subsequent structural and functional changes in V1b [1]. MD during the critical period decreases the strength of deprived eye responses and the density of spines on the apical dendrites of supragranular neurons [2,3,4]. An extension of the MD duration reveals the potentiation of non-deprived inputs, enabled by deprived eye depression [5] and accompanied by a recovery of dendritic spine density [3]. Changes in spatial acuity induced by brief MD during the critical period can be reversed rapidly [6,7,8]. In contrast, chronic MD (cMD) induced prior to the onset of the critical period and maintained until adulthood induces a significant reduction in the spatial acuity of the chronically-deprived eye, which is highly resistant to recovery [9,10,11].

### Extracellular Proteolysis Enables Plasticity in Immature and Mature Circuits

We have previously shown that the visual deficits induced by cMD can be reversed in adults by dark exposure followed by light reintroduction [11]. Activation of extracellular proteolysis has been repeatedly shown to reactivate synaptic plasticity in the adult brain [12,13,14]. Accordingly, an enzyme that degrades the chondroitin sulfate proteoglycans of the extracellular matrix (ECM) reactivates robust ocular dominance plasticity in adult rat V1 [15,16]. Similarly, hyaluronidase, which cleaves the hyaluronic acid backbone of ECM, enhances short-term plasticity in mature cultured neurons and rescues plasticity in adult MMP9^−/−^ mice [17,18]. Other manipulations that attenuate the perineuronal net specializations of the ECM, such as deletion of cartilage link protein, eliminate the constraint on ocular dominance plasticity with age [19].

MMP9 is of particular interest, as MMP9 activity has been repeatedly shown to couple synaptic activity to structural plasticity in both mature and immature circuits. In the adult hippocampus, theta burst stimulation increases MMP9 activity, and MMP9 inhibition blocks the maintenance of synaptic potentiation and several forms of learning [20,21,22,23]. In cultured hippocampal neurons, the pharmacological induction of synaptic potentiation increases MMP9 activity, specifically at enlarging or emerging spines [24]. In hippocampal slices from young rats, the application of MMP9 is sufficient to potentiate the size and strength of CA3-CA1 excitatory synapses and enable plasticity onto CA2 neurons [25,26]. Many manipulations, including the administration of anti-depressants, reveal an inverse correlation between MMP9 activity and the density of perineuronal net on inhibitory neurons [27]. However, activation of MMP9 has also been implicated in synaptic weakening and may represent an obligatory step for many forms of structural and functional plasticity [28,29]. The reactivation of structural and functional plasticity in adult mouse V1 by light reintroduction following dark exposure requires an increase in activity of endogenous MMP9 [18]. The threshold for visually-evoked activation of perisynaptic MMP9 is regulated by visual experience and lowered by dark exposure at thalamo−cortical, but not cortico−cortical, synapses in adult V1 [30].

Here, we ask if deletion of MMP9 impacts neuronal response properties in binocular and chronically monocularly deprived adults. We find that visual response strength and selectivity is normal in adult MMP9^−/−^ mice. However, the changes in neuronal response strength and selectivity induced by cMD in wild-type (WT) mice are absent in MMP9^−/−^ mice.

## 2. Results

### 2.1. cMD Decreases the Strength of the Deprived Eye Pathway of WT but Not MMP9^−/−^ Mice

**Figure 1:**
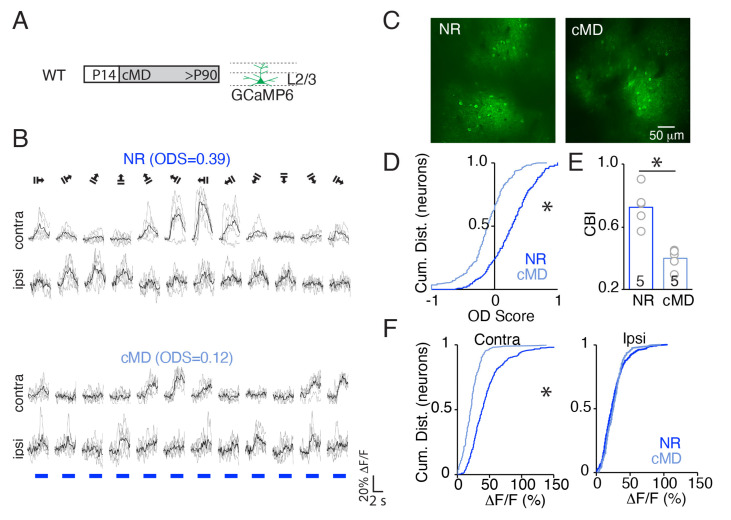
cMD decreases the strength of the deprived eye pathway in WT but not MMP9^−/−^ mice. (**A**) Left: Experimental timeline. MD was initiated at eye opening (postnatal day 14 (P14)) and maintained until adulthood (>P90). Right: GCaMP6s expression was targeted to layer 2/3 neurons of WT V1b (AAV1.hSyn1.mRuby2.GSG.P2A.GCaMP6s.WPRE.SV40, Addgene, titer: 1.3 × 10^13^ U/mL, 30 nl, AP: 1.0 mm, MD: −3.0 mm, DV: 0.3 mm; at least 3 weeks prior to imaging) to monitor visual evoked calcium transients. (**B**) Representative ΔF/F of the GCaMP6s signal evoked in layer 2/3 of V1b in normal reared (NR) and cMD WTs in response to the presentation of drifting square wave gratings (0.05 cycle/degree, 100% contrast, 28 cd/m^2^ at 12 orientations) to contra- and ipsilateral eyes. Blue bar = stimulus onset. Individual trials in grey, average of 5 repeats in black. Ocular dominance score (ODS) = (C − I)/(C + I) was calculated at the preferred orientation for each neuron. (**C**) Examples of two-photon field views of GCaMP6s expression. (**D**) Cumulative distribution of ODS. * *p* = 1.0 × 10^−4^, KS test. (**E**) Mean CBI of layer 2/3 neurons is significantly lower in cMD than NR WT mice. * *p* = 0.012, Mann−Whitney test. *n* = 5 subjects. (**F**) Cumulative distribution of contra and ipsi eye neuronal responses (ΔF/F; * *p* = 8.3 × 10^−29^ for contra, *p* = 0.91 for ipsi, Student’s *t*-test, 365 and 201 neurons for NR and cMD, respectively).

The critical period for ocular dominance plasticity peaks during the third postnatal week in mice, one week after eye opening. To quantify the impact of a long-term asymmetry in the quality of vision across the two eyes initiated immediately after eye opening, we measured visually-evoked calcium transients in layer 2/3 neurons of awake adult C57BL/6J mice (WT) following chronic monocular deprivation from P14 until adulthood (>P90, up to P170). Image acquisition was restricted to 150 to 250 μm from the brain surface to focus on neuronal cell bodies in layer 2/3 in the binocular region of the primary visual cortex (V1b) following targeted delivery of GCaMP6s (AAV1.hSyn1. mRuby2.GSG.P2A.GCaMP6s.WPRE.SV40, Addgene, titer: 1.3 × 10^13^ U/mL, 30 nl, Figure 1A).

Visually-evoked calcium transients in response to drifting square wave gratings (0.05 cycle/degree (cpd),100% contrast) in 12 directions were analyzed to identify the neuronal response preferences in normal-reared (NR) adult (>P90) wild type (WT) subjects (Figure 1B). The mean ΔF/F in response to the preferred stimulus was 46 ± 2%, which is predicted to translate to 5–6 action potentials [31] and is consistent with previous reports of visually-evoked firing rates in V1 of adult mice [32,33,34]. The majority of neurons were binocular (ocular dominance score (ODS) = (C − I)/(C + I)), ODS ≠ ±1) and preferred contralateral (ODS > 0.2) eye stimulation (Figure 1C,D). cMD decreased the preference for contralateral eye stimulation (mean ΔF/F: 22 ± 1%), and induced a significant leftward shift in the cumulative distribution of neuronal ODS (mean ± SEM: 0.26 ± 0.02 for NR, −0.13 ± 0.02 for cMD, * *p* = 1.0 × 10^−4^, KS Test, Figure 1D) and the average contralateral bias index (CBI; see methods) across subjects (mean ± SEM: NR 0.73 ± 0.05 for NR, cMD 0.40 ± 0.03 for cMD, ** *p* = 0.012, Mann−Whitney test, *n* = 5 subjects, Figure 1E).

Monocular visual responses (ΔF/F) following cMD revealed the strength of response to the stimulation of the deprived, contralateral eye was significantly reduced but the strength of response to the stimulation of the non-deprived, ipsilateral eye was unchanged (mean ± SEM: 46 ± 2% for NR, 22 ± 1% for cMD, Student’s *t*-test, * *p* = 8.3 × 10^−29^ for contra, 26 ± 1% for NR, 26 ± 1% for cMD, *p* = 0.91 for ipsi, 365 and 201 neurons for NR and cMD, respectively, Figure 1F). Thus, the response to cMD differs from the response to MD initiated during the peak of the critical period, which is biphasic and includes strengthening of the non-deprived input [2,35].

**Figure 2:**
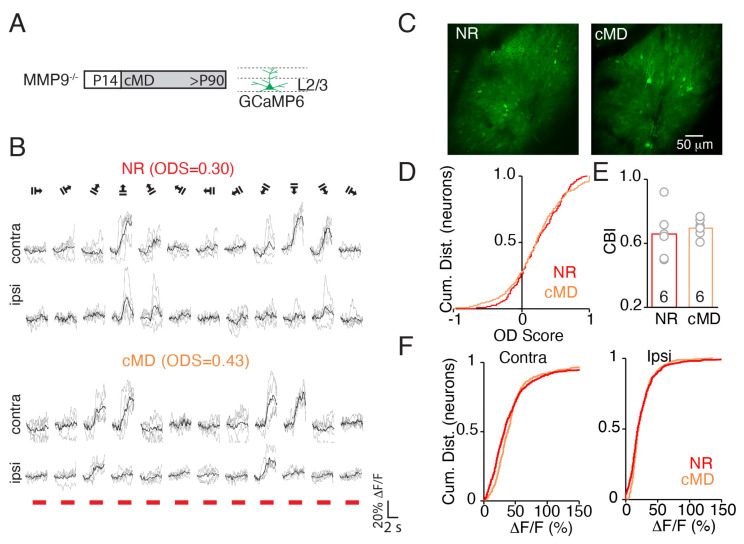
Ocular dominance in MMP9^−/−^ mice is normal and resistant to cMD. (**A**) Left: Experimental timeline. Right: GCaMP6s expression was targeted to layer 2/3 neurons of MMP9^−/−^ V1b. (**B**) Representative ΔF/F of GCaMP6 signal evoked in layer 2/3 of V1b in normal reared (NR) and cMD MMP9^−/−^ mice in response to presentation of drifting square wave gratings (0.05 cycle/degree, 1 Hz, 100% contrast, 28 cd/m^2^ at 12 orientations) to contra- and ipsilateral eyes. Red bar = stimulus onset. Individual trials in grey, average of 5 repeats in black. Ocular dominance score (OD score) = (C − I)/(C + I) was calculated at the preferred orientation for each neuron. (**C**) Examples of two-photon field views of GCaMP6s expression. (**D**) Cumulative distribution of ODS (*p* = 0.42, KS test). (**E**) Mean CBI of layer 2/3 neurons is similar in cMD and NR MMP9^−/−^ mice. *p* = 0.38, Mann−Whitney test. *n* = 6 subjects. (**F**) Cumulative distribution of contra and ipsi eye visual responses (ΔF/F; *p* = 0.75 for contra, *p* = 0.63 for ipsi, Student’s *t*-test, 328 and 595 neurons for NR and cMD, respectively).

In MMP9^−/−^ mice, the expression of GCaMP6s and the neuronal response preferences in normal-reared (NR) adult (>P90) subjects were comparable to WTs (Figure 2A,B). Similarly, the map of ODS of each visually-responsive neuron in V1b revealed a “salt and pepper” distribution of eye preference. Again, we found that the majority of neurons were binocular (ODS ≠ ±1) and preferred contralateral (ODS > 0.2) eye stimulation (Figure 2C,D). This is consistent with our previous observation that the visual system is grossly normal following the deletion of MMP9 [18]. However, in MMP9^−/−^ mice, cMD did not decrease the preference for contralateral eye stimulation, and retained a normal cumulative distribution of neuronal ODS (mean ± SEM: 0.23 ± 0.02 for NR, 0.21 ± 0.02 for cMD, *p* = 0.42, KS Test, Figure 2D) and normal average CBI across subjects (mean ± SEM: 0.66 ± 0.06 for NR, 0.70 ± 0.02 for cMD, *p* = 0.38, Mann−Whitney test, *n* = 6 subjects, Figure 2E). Accordingly, cMD did not reduce the strength of response to stimulation of the deprived contralateral or non-deprived ipsilateral eye (ΔF/F, mean ± SEM: 49 ± 3% for NR, 48 ± 2% for cMD, Student’s *t*-test, *p* = 0.74, for contra, 26 ± 1% for NR, 26 ± 1% for cMD, *p* = 0.42, for ipsi, *n* = 328 and 595 neurons for NR and cMD, respectively, Figure 2F). Thus, the ocular preference of MMP9^−/−^ mice is resistant to prolonged monocular deprivation initiated at eye opening and maintained until adulthood.

**Figure 3:**
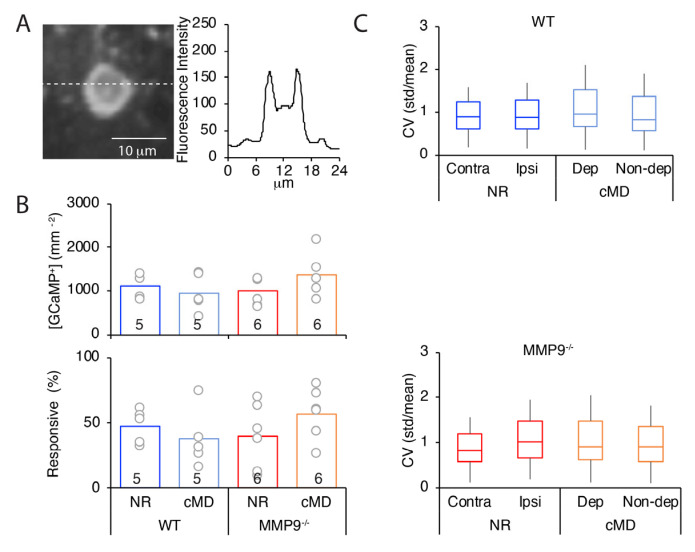
Stability of GCaMP6s expression and visually responsive neurons over experimental conditions. (**A**) Left: representative neuron from layer 2/3 of V1b expressing GCaMP6s. Right: Quantification of fluorescence along dashed white line reveals a high signal in the cytoplasm relative to the nucleus. (**B**) Top: No difference in the total number of GCaMP6s expressing neurons between WT and MMP9^−/−^, normal reared (NR) and cMD subjects (one-way ANOVA, F = 3.0, *p* = 0.06, *n* = 5 and 6 subjects for WT and MMP9^−/−^ mice, respectively). Bottom: Percent of GCaMP6s expressing neurons that are visually-responsive (one-way ANOVA, F = 0.96, *p* = 0.43). A neuron is defined as visually responsive if the mean ΔF value in response to a visual stimulus of any direction exceeds 3 × STD of baseline (F_0_) for > 40% of trials during either contra or ipsi eye stimulation. (**C**) Coefficient of variance (CV) of calcium transients (standard deviation over mean ΔF/F (STD/mean) is comparable across all experimental conditions, and is unaffected by the deletion of MMP9 or cMD. Box plots represent the median as a bar, 25th to 75th percentile as box, and max and min as whiskers (WT: one-way ANOVA, F = 7.1, *p* = 0.10, *n* = 359, 364, 201, and 194 neurons for NR contra, NR ipsi, cMD deprived, and cMD non-deprived, respectively; MMP9^−/−^: one-way ANOVA, F = 4.3, *p* = 0.11, *n* = 315, 321, 595, and 595 neurons for NR contra, NR ipsi, cMD deprived, and cMD non-deprived, respectively).

**Figure 4:**
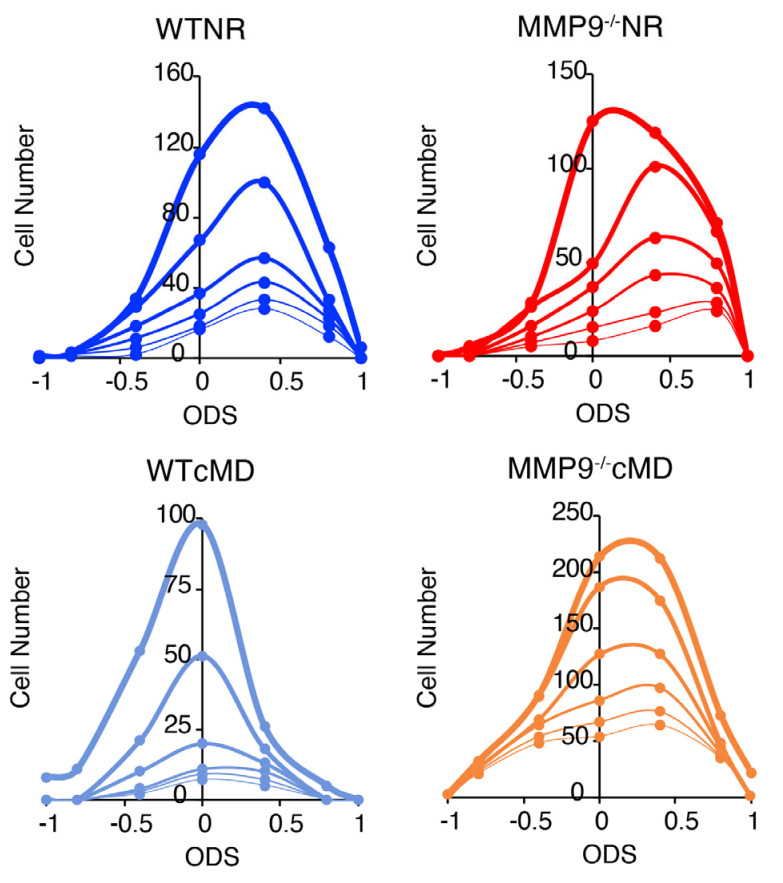
Distributions of ocular dominance scores are independent of the criterion for neuronal visual responsivity. Number of neurons in different ODS categories (ODS = 1, 1 > ODS ≥ 0.6, 0.6 > ODS ≥ 0.2, 0.2 > ODS ≥ −0.2, −0.2 > ODS ≥ −0.6, −0.6 > ODS ≥ −1, and ODS = −1) following different inclusion criteria for visually-responsive neuronal activity (thickest to thinnest lines: 3×, 4×, 5×, 6×, 7×, and 8× STD of baseline).

Neither the genotype nor visual experience impacted the GCaMP6s expression or neuronal visual responsivity. In all cases, calcium imaging was performed in adult mice (>P90, up to P170), at least 3 weeks after the delivery of GCaMP6s to the mouse V1b, following confirmation of high cytosolic expression excluded from the nucleus (Figure 3A [36]). Neither the deletion of MMP9 nor cMD changed the number of neurons expressing GCaMP6s (GCaMP^+^ neurons: 1122.0 ± 112.9 mm^−2^ for NR WT, 958.4 ± 193.1 mm^−2^ for cMD WT, 992.2 ± 123.6 mm^−2^ for NR MMP9^−/−^, 1353.8 ± 202.3 mm^−2^ for cMD MMP9^−/−^; one-way ANOVA, F = 0.67, *p*= 0.58, *n* = 5 and 6 subjects for WT and MMP9^−/−^ mice, respectively, Figure 3B), nor the % of GCaMP-expressing neurons in mouse V1b that were visually-responsive defined as mean ΔF value in response to any of the 12 visual stimuli > 3 × STD of baseline (F_0_) for > 40% or trials (47.5 ± 5.8% for NR WT, 37.7 ± 10.0% for cMD WT, 40.1 ± 9.3% for NR MMP9^−/−^, 57.2 ± 8.9% for cMD MMP9^−/−^, one-way ANOVA, F = 0.96, *p*= 0.43, *n* = 5 and 6 subjects for WT and MMP9^−/−^ mice, respectively, Figure 3B). The coefficient of variance (CV) of calcium transients (standard deviation over mean ΔF/F (std/mean)) was also comparable across all imaging conditions (one-way ANOVA, F = 7.1, *p* = 0.10, *n* = (359, 364, 201, and 194 neurons) for WTNR contra, WTNR ipsi, WTcMD deprived, and WTcMD non-deprived eye, respectively (F = 4.3, *p* = 0.11, *n* = 315, 321, 595, and 595 neurons) for MMP9^−/−^NR contra, MMP9^−/−^NR ipsi, MMP9^−/−^cMD deprived, and MMP9^−/−^cMD non-deprived eye, respectively; Figure 3C). Furthermore, the initial preference of binocular neurons for contralateral eye stimulation and the reduction in contralateral bias induced by cMD was observed across a range of inclusion criteria for visually-responsive neurons (Figure 4).

### 2.2. cMD Decreases the Strength, but Not the Selectivity of the Deprived Eye Pathway of WT but Not MMP9^−/−^ Mice

**Figure 5:**
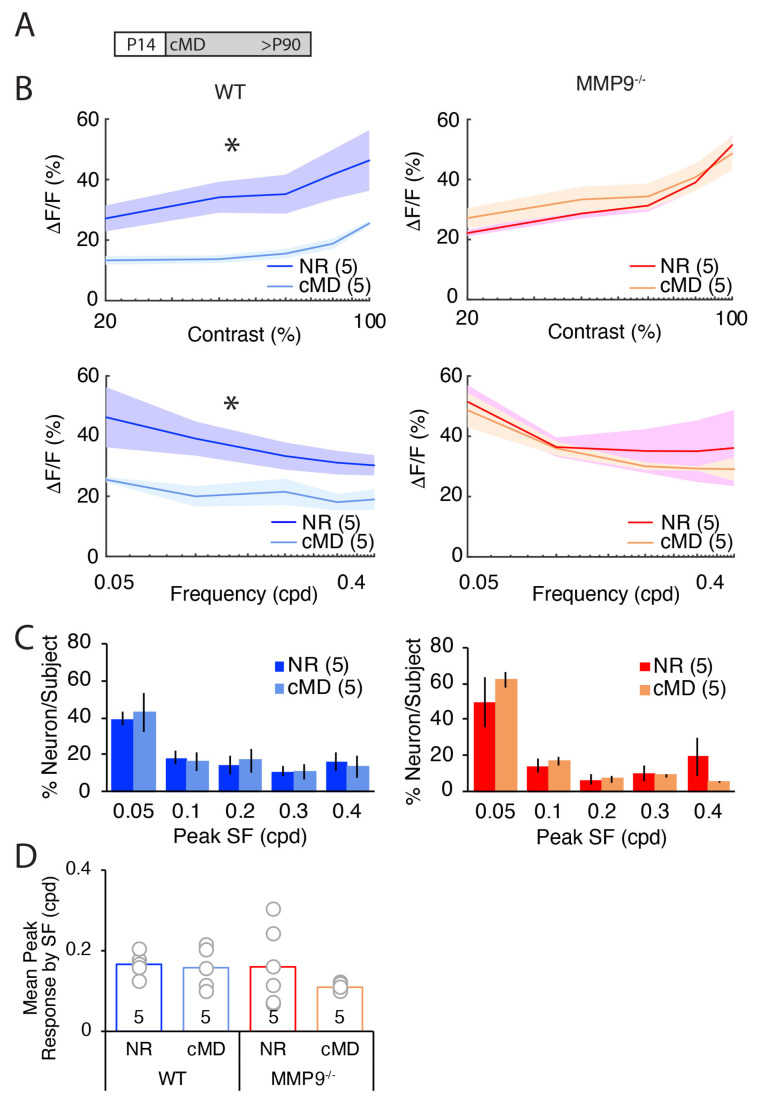
cMD impairs the strength of visual responses across contrasts and spatial frequencies in WT, but not MMP9^−/−^ mice. (**A**) Experimental paradigm. (**B**) Significant decrease in the strength of neuronal responses to a range of contrasts (top) and spatial frequencies (bottom) in cMD versus NR WT (left), but not in MMP9^−/−^ (right) mice. Mean ΔF/F values of responsive neurons are plotted for NR (dark blue) and cMD (light blue) for WT, NR (red) and cMD (orange) for MMP9^−/−^ (repeated measures ANOVAs: contrast at 0.05 cpd, WT F = 9.7, * *p* = 0.014, MMP9^−/−^ F = 0.20, *p* = 0.67; spatial frequency at 100% contrast: WT F = 8.3, * *p* = 0.020, MMP9^−/−^ F = 0.33, *p* = 0.58; *n* = 5 subjects). (**C**) Distribution of the spatial frequency evoking the peak neuronal response. Right: NR WT (dark blue) and cMD WT (light blue). Left: NR MMP9^−/−^ (red) and cMD MMP9^−/−^ (orange). Mean ± SEM, *n* = 5 subjects. (**D**) Mean peak spatial frequency is similar in cMD and NR in both WT and MMP9^−/−^ mice (one-way ANOVA, F = 0.89, *p* = 0.47, *n* = 5 subjects).

To quantify the impact of cMD on the selectivity of neurons in mouse V1b for visual stimulus spatial frequency and contrasts, we presented low spatial frequency stimuli (0.05 cycle/degree (cpd)) at contrasts ranging from 20–100%, and high contrast stimuli (100%) at spatial frequencies ranging from 0.05 to 0.4 cpd. In NR adult WTs, the magnitudes of evoked calcium transients were positively correlated with visual stimulus contrasts (y = 11 ln(x) − 7, R^2^ = 0.92; Figure 5, top left dark blue). cMD significantly reduced the magnitude of the visually-evoked response across the range of contrasts (repeated measures ANOVA, F = 9.7, * *p* = 0.014, *n* = 5 subjects; Figure 5B top left light blue). In NR adult MMP9^−/−^ mice, response magnitude and stimulus contrast were also positively correlated (y = 16 ln(x) − 29, R^2^ = 0.82) and comparable to NR WT, as predicted (Figure 5B top right red [18]). However, cMD did not impact the amplitude of the evoked response at any visual stimulus contrast (repeated measures ANOVA, F = 0.20, *p* = 0.67, *n* = 5 subjects; Figure 5B top right orange).

Similarly, in NR WT mice, the response magnitude was negatively correlated with the visual stimulus spatial frequency (y = −8 ln(x) + 22, R^2^ = 0.98; Figure 5B bottom left dark blue). cMD significantly reduced the magnitude of the visually-evoked response across the range of spatial frequencies (repeated measures ANOVA, F = 8.3, * *p* = 0.02, *n* = 5 subjects; Figure 5B bottom left light blue). In NR adult MMP9^−/−^ mice, response magnitude and visual stimulus spatial frequency were also negatively correlated (y = −9 ln(x) + 18, R^2^ = 0.88) and comparable to WTs (Figure 5B bottom right red). Again, cMD did not impact the magnitude of the evoked responses at any stimulus spatial frequency (repeated measures ANOVA, F = 0.33, *p* = 0.58, *n* = 5 subjects; Figure 5B bottom right orange).

Despite the significant decrease in amplitude of visually-evoked responses at all SFs in WTs following cMD, SF tuning was unchanged, with ~40% of neurons in both NR and cMD conditions preferring 0.05 cpd visual stimuli (Figure 5C). A similar preference for low spatial frequency visual stimuli was observed in MMP9^−/−^ mice, which was unchanged by cMD (Figure 5C). Accordingly, the mean peak neuronal response binned by SF did not differ across the genotypes or visual history (mean ± SEM: 0.17 ± 0.01 cpd, 0.16 ± 0.02 cpd, 0.16 ± 0.04 cpd, 0.11 ± 0.003 cpd for NR WT, cMD WT, NR MMP9^−/−^, cMD MMP9^−/−^, respectively, one-way ANOVA, F = 0.89, *p* = 0.47, *n* = 5 subjects; Figure 5D). Thus, cMD impacts the strength, but not the selectivity, for visual stimulus spatial frequency and contrast in WT, but not MMP9^−/−^ mice.

### 2.3. cMD Impacts the Strength and Selectivity to Visual Stimulus Orientation and Direction in WT but Not MMP9^−/−^ Mice

**Figure 6:**
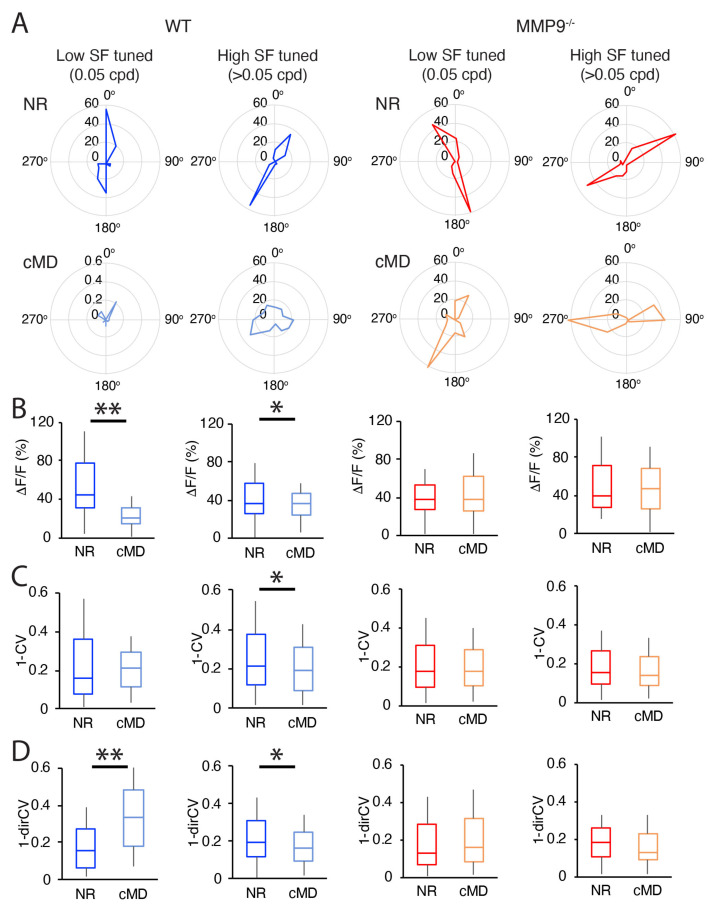
Orientation and direction selectivity are impacted by cMD in WT but not MMP9^−/−^ mice. (**A**) Representative polar plots of low spatial frequency (SF) tuned (0.05 cpd) and high SF tuned (>0.05 cpd) neurons of WT and MMP9^−/−^ mice. NR WT (dark blue) and cMD WT (light blue), NR MMP9^−/−^ (red), and cMD MMP9^−/−^ (orange). Scale: ΔF/F (%). (**B**) cMD significantly decreased ΔF/F in WT but not MMP9^−/−^ mice. (**C**) Orientation selectivity (1-CV) in high SF tuned (>0.05 cpd) neurons is lower in cMD (light blue) than NR (dark blue) WT (left) but not MMP9^−/−^ (right, red for NR and orange for cMD) mice. (**D**) Direction selectivity (1-dirCV) is higher in low SF tuned neurons (0.05 cpd) in cMD (light blue) than NR (dark blue), and lower in high SF tuned (>0.05 cpd) neurons in cMD WT, but not in MMP9^−/−^ mice. Box plots represent median as a bar, 25th to 75th percentile as box, and max and min as whiskers, * *p* < 0.05, ** *p* < 0.001, Student’s *t*-test, *n* = 59, 51, 94, 95, 208, 88, 104, and 160 neurons for LSF NR WT, LSF cMD WT, HSF NR WT, HSF cMD WT, LSF NR MMP9^−/−^, LSF cMD MMP9^−/−^, HSF NR MMP9^−/−^, and HSF cMD MMP9^−/−^, respectively.

In mouse V1b, eye preference co-varies with spatial frequency tuning and selectivity for visual stimulus direction and orientation [37]. We therefore asked if cMD impacted the orientation or direction selectivity of V1b neurons tuned to distinct spatial frequencies in WT and MMP9^−/−^ mice. Representative polar plots revealed the strength and selectivity of visual responses for drifting gratings in the two genotypes (Figure 6A). In WT mice, cMD induced a significant decrease in the amplitude of visually-evoked ΔF/F in neurons tuned to either low spatial frequencies (LSF: 0.05 cpd: mean ± SEM: 57 ± 5%, 25 ± 3%, *n* = 59, 51 neurons; 62% and 54% of total; 3 and 4 subjects for NR WT and cMD WT, respectively, ** *p* < 0.001, Student’s *t*-test) and high SFs (>0.05 cpd; mean ± SEM: 46 ± 3%, 38 ± 2%, *n* = 94 and 95 neurons for NR WT and cMD WT, respectively, * *p* < 0.05, Student’s *t*-test). In contrast, in MMP9^−/−^ mice, no change in visual response strength (ΔF/F) was observed in neurons tuned to either low SF (mean ± SEM: 46 ± 3%, 55 ± 5%, *n* = 208, 88 neurons; 67% and 65% of total; 3 and 3 subjects for NR and cMD, respectively, Figure 6B), or high SFs following cMD (mean ± SEM: 53 ± 4%, 56 ± 4%, *n* = 104, 160 NR, cMD, respectively, Figure 6B). Interestingly, in WTs, cMD reduced the orientation selectivity (1−CV) in the neurons tuned to high (>0.05 cpd) but not low SFs (<0.05 cpd; mean ± SEM: 0.25 ± 0.03, 0.29 ± 0.04, 0.28 ± 0.02, 0.21 ± 0.02, *n* = 59, 51, 94, and 95 neurons for LSF NR, LSF cMD, HSF NR, and HSF cMD, respectively, * *p* < 0.05, Student’s *t*-test). Again, no change in orientation selectivity was observed following cMD in MMP9^−/−^ mice (mean ± SEM: 0.25 ± 0.02, 0.24 ± 0.02, 0.20 ± 0.02, and 0.19 ± 0.01, *n* = 208, 88, 104, and 160 neurons for LSF NR, LSF cMD, HSF NR, and HSF cMD, respectively; Figure 6C). cMD increased the direction selectivity (1−dirCV) of the neurons tuned to low SFs in WT, but not MMP9^−/−^ mice (mean ± SEM: 0.21 ± 0.03, 0.38 ± 0.04, 0.22 ± 0.02, and 0.23 ± 0.02, *n* = 59, 51, 208, and 88 neurons for LSF NR WT, LSF cMD WT LSF NR MMP9^−/−^, and LSF cMD MMP9^−/−^, respectively; Figure 6D). Conversely, cMD decreased the direction selectivity of the neurons turned to high SFs (>0.05 cpd) in WT but not MMP9^−/−^ mice (mean ± SEM: WT 0.27 ± 0.02, 0.20 ± 0.02, *n* = 94 and 95 neurons for NR and cMD, respectively; MMP9^−/−^ 0.20 ± 0.01, 0.19 ± 0.01, *n* = 104 and 160 neurons for NR and cMD MMP9^−/−^, respectively; Figure 6D). Thus, the plasticity of neuronal selectivity for orientation and direction engaged by cMD in WTs was not observed in MMP9^−/−^ mice.

### 2.4. Anatomical Correlates of cMD in WT and MMP9^−/−^ Mice

To examine the impact of experience-dependent development and plasticity upstream of V1, we traced the distribution of LGN axon terminals in the deprived and non-deprived mice. Intraocular injection of fluorophore-conjugated cholera toxin subunit B (CTB) revealed the expected eye-specific segregation in both normal-reared (NR) WT and MMP9^−/−^ mice (ipsi occupancy of mean ± SEM: 16.9 ± 1.5% for right eye (RE) of WT NR, 15.2 ± 1.8% for RE of MMP9^−/−^ NR, *p* = 0.49, Student’s *t*-test, *n* = 4 subjects, Supplemental Figure S1A; [38,39,40,41,42]). cMD did not grossly impair eye-specific RGC segregation in the dLGN of either genotype (ipsi occupancy mean ± SEM: 16.9 ± 1.5% for RE of WT NR, 17.5 ± 1.0% for deprived eye (dep) of cMD, *p* = 0.74; 15.2 ± 0.9% RE of MMP9^−/−^ NR, 15.3 ± 1.5% for dep of cMD, *p* = 0.98; Student’s *t*-test, *n* = 4 subjects, Supplemental Figure S1A).

We quantified the number and size of the vesicular glutamate transporter (VGluT2) puncta in thalamic axons to assess the impact of cMD on the feedforward input from dLGN to layer 4 (Supplemental Figure S1B). cMD significantly lowered the density of VGluT2 puncta in deprived (dep) versus non-deprived (non) V1b in WTs (mean ± SEM: 203.4 ± 25.4/0.01 mm^−2^ for dep, 395.0 ± 49.8/0.01 mm^−2^ for non, Paired *t*-test, * *p* = 0.048, *n* = 5 subjects Supplemental Figure S1B), similar to changes induced by brief MD during the peak of the critical period [43]. However, the size of VGluT2 puncta that persisted following cMD were comparable to normal (mean ± SEM: size 0.34 ± 0.007 μm^2^ for dep, 0.37 ± 0.01 μm^2^ for non, Paired *t*-test, *p* = 0.15; intensity 46.7 ± 7.3 pixel for dep, 53.4 ± 3.8 pixel for non, Paired *t*-test, *p* = 0.51; *n* = 5 subjects; Supplemental Figure S1B). In contrast, cMD did not impact the density or size of VGluT2 puncta in MMP9^−/−^ mice (mean density ± SEM: 281.9 ± 21.0/0.01 mm^−2^ for dep, 243.3 ± 44.9/0.01 mm^−2^ for non, Paired *t*-test, *p* = 0.37, *n* = 6 subjects, mean size ± SEM: 0.34 ± 0.007 μm^2^ for dep, 0.37 ± 0.01 μm^2^ for non, Paired *t*-test, *p* = 0.26, *n* = 6 subjects, mean intensity ± SEM: 63.6 ± 6.1 pixel for dep, 63.7 ± 4.8 pixel for non, Paired *t*-test, *p* = 0.96, *n* = 6 subjects).

We have previously reported a significant reduction in dendritic spines in thalamo-recipient layer 4 of rat visual cortex following cMD [10]. To ask if this morphological plasticity was dependent on MMP9, we compared the density and diameter of dendritic spines of basolateral dendrites of V1b in layer 4 neurons following cMD in WT and MMP9^−/−^ mice. The analysis was limited to dendritic segments 75–100 microns from the pyramidal neuron cell body (Supplemental Figure S1C, [44,45]), the target of axons from the dorsolateral geniculate nucleus core [46]. In WTs, cMD induced a significant decrease in the density of basolateral dendritic spines of layer 4 neurons in deprived (dep) relative to non-deprived (non) V1b (mean ± SEM: 1.12 ± 0.08 μm^−1^ for non, 0.77 ± 0.06 for dep, KS test, *p* = 7.3 × 10^−4^, *n* = 20 neurons each from 5 subjects, Supplemental Figure S1C). However, the diameter of dendritic spines that persisted following cMD were comparable to NR (mean ± SEM: 0.72 ± 0.03 μm for non, 0.71 ± 0.02 μm for dep, KS test, *p* = 0.99, *n* = 20 neurons each from 5 subjects; Supplemental Figure S1C). No changes in the dendritic spine density of the diameter were observed in non-deprived V1b following cMD.

As expected, in MMP9^−/−^ mice, the initial spine density of layer 4 neurons was lower than the WT juveniles or adults, but the spine diameters were normal [18,47]. However, cMD had no impact on dendritic spine density or spine morphology (mean ± SEM density: 0.66 ± 0.08 μm^−1^ for non, 0.70 ± 0.08 μm^−1^ for dep, KS test, *p* = 0.0014; diameter: 0.70 ± 0.02 μm for non, 0.72 ± 0.02 μm for dep, KS test, *p* = 0.80, *n* = 18 neurons for non, 19 neurons for dep from 4 subjects; Supplemental Figure S1C), indicating the absence of the morphological plasticity of thalamocortical synapses engaged by cMD MMP9^−/−^ mice.

### 2.5. Persistence of Stimulus-Selective Response Potentiation (SRP) in Adult MMP9^−/−^ Mice

**Figure 7:**
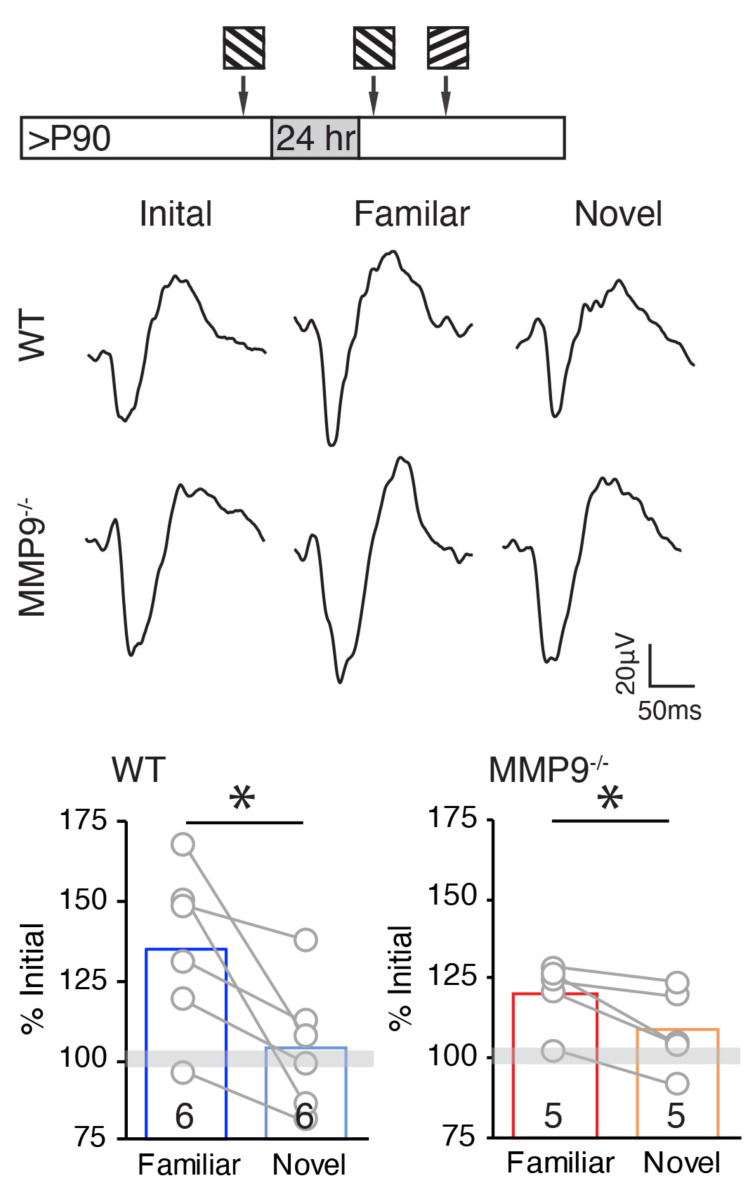
Stimulus-Selective Response Potentiation persists in adult MMP9^−/−^ mouse V1b. Top: Experimental timeline. Middle: Representative layer 4 VEPs of WT and MMP9^−/−^ mice in response to the initial 200 presentations of visual stimulation at a single orientation (0.05 cpd 100% contrast gratings reversing at 1 Hz), and a familiar and novel stimuli presented 24 h later. Bottom: Mean VEP amplitudes in WT and MMP9^−/−^ mice. Grey bar = mean ± SEM of initial VEP; 24 h later, a significant increase in the VEP amplitude is observed in response to the familiar (left) but not novel (right) stimulus orientation, in both WT and MMP9^−/−^ mice, * *p* < 0.05, Student’s *t*-test. *n* = 6 and 5 subjects for WT and MMP9^−/−^, respectively.

Finally, we asked in the visual system of MMP9^−/−^ adult mice can express other types of experience-dependent plasticity. Repetitive visual stimulation is known to induce a robust stimulus-selective response potentiation (SRP) in mouse V1, via a mechanism that is distinct from that engaged by monocular deprivation [48,49]. Accordingly, repetitive visual stimulation (200 presentations of 100% contrast square wave gratings, 0.05 cpd, reversing at 1 Hz) induced a significant increase in the amplitude of layer 4 visually-evoked potentials (VEPs) in response to a familiar, but not novel, visual stimulus orientation (mean ± SEM: familiar: 135.4 ± 10.5% of initial (grey bar), * *p* = 0.04, Student’s *t*-test, novel: 103.7 ± 8.4% of initial, *p* = 0.36, Student’s *t*-test, *n* = 6 subjects; Figure 7). Importantly, repetitive visual stimulation induced a similar potentiation of the layer 4 VEP in response to familiar, but not novel visual stimulus (mean ± SEM: familiar: 120.4 ± 4.7% of initial, * *p* = 0.03, novel: 108.7 ± 5.8% of initial, *p* = 0.16, Student’s *t*-test, *n* = 5 subjects; Figure 7), demonstrating that the visual cortex of MMP9^−/−^ mice retained the ability to express robust SRP.

## 3. Discussion

### 3.1. cMD Regulates Visual Response Strength and Selectivity in WT but Not MMP9^−/−^ Mice

We demonstrate that manipulations of postnatal visual experience disassociate MMP9-dependent and -independent forms of plasticity in the murine visual system. In WT mice, chronic monocular deprivation from eye opening to adulthood reduces the strength of deprived eye visually-evoked responses to all stimulus spatial frequencies and contrasts. Furthermore, cMD impacts the neuronal selectivity for orientation and direction, but not spatial frequency. Constitutive deletion of MMP9 did not impact the stimulus selectivity of V1b neurons, including ocular preference and tuning for spatial frequency, orientation, and direction. However, cMD did not impact the strength or the tuning of the neuronal responses evoked by deprived eye stimulation. Nonetheless, other forms of activity-dependent plasticity persist in MMP9^−/−^ mice, including eye specific segregation of RGC axons in the dLGN, the expression and maintenance of contralateral bias of visually-evoked responses, and robust stimulus selective response potentiation induced by repetitive visual stimulation. Thus, MMP9 is obligatory for the plasticity engaged by cMD at eye-opening, but is dispensable for many other forms of plasticity that refine and maintain the mouse visual system.

### 3.2. cMD and MD during the Critical Period Recruit Distinct Forms of Plasticity

Changes in visual response strength induced by cMD were limited to deprived eye responses, and therefore differed from the response to MD at the peak of the critical period, in which deprived eye depression is followed by an increase in the strength and selectivity of non-deprived eye responses [2,35,50,51]. The decrease in the strength of visual responses following cMD was observed across a range of spatial frequencies and contrasts. The absence of a change in SF tuning selectivity following cMD was unexpected, given the significant reduction in deprived eye spatial acuity demonstrated by performance in a visual discrimination task [52]. A disassociation between spatial frequency tuning of individual V1 neurons and spatial acuity has also been described in strabismic macaques [53,54,55].

Tuning for SF is broader than tuning for orientation and direction [56,57]. Although the tuning for different aspects of the visual stimulus can be independently regulated [58], tuning to high spatial frequencies is associated with contralateral eye dominance [37]. cMD decreased orientation and direction selectivity in neurons tuned to high spatial frequencies, indicating co-regulation of eye preference, orientation and direction selectivity by cMD. In contrast, the increase in direction selectivity in neurons tuned to low spatial frequencies observed following cMD is predicted by the increase in binocularity [59].

### 3.3. Assessment of Binocularity in Mouse V1b

The characterization of neuronal responses in mouse V1b as binocular has recently come under scrutiny [37,59,60,61,62]. Central to this debate is the role of inclusion criteria in the definition of visually-evoked GCaMP activity, as the adaptation of high-threshold inclusion criteria excludes contributions from the non-dominant eye [51]. However, in our hands, increasing the threshold for the inclusion of visually-evoked activity from the non-dominant eye did not reduce the number of neurons categorized as binocular in any experimental populations of adult mice. Our classification of the majority of visually evoked responses in V1b as binocular is consistent with a large body of work utilizing single unit recordings to measure spiking output in response to contra and ipsilateral eye stimulation [63,64,65,66,67,68]. The expression of binocularity in mouse V1b may also be impacted by the strategy for the expression of GCaMP.

### 3.4. Structural and Functional Plasticity Induced by MMP9 Activity

Although MMP9 activity is confined to excitatory synapses, it is differentially expressed across synapse classes in adult mouse V1b. The baseline MMP9 activity is high at cortico-cortical relative to thalamo-cortical synapses, which may contribute to differences in the rates of dendritic spine turnover [18,69]. Importantly, light reintroduction after dark exposure increases the activity of MMP9 preferentially at thalamocortical synapses in adult V1b, and the threshold for the induction of MMP9 activity at these synapses is regulated by the visual experience [30].

The targets of MMP9 that mediate the response to manipulations of visual experience have yet to be defined, but include a number of perisynaptic extracellular molecules that regulate synaptic plasticity [25,70,71]. Potential targets include the dendritic cell adhesion molecule telencephalin [72], which negatively regulates spine maturation [73]. MMP9 deletion prevents the translocation of telencephalin from spine heads to shafts over development [74]. Telencephalin cleavage following tetanic simulation or NMDAR activation, and the spine enlargement that is characteristic of long-term potentiation, are blocked by MMP inhibition [71,75]. However, the long, thin spines characteristic of mouse model of fragile-X-mental-retardation (FMR1^−/−^ mice) are normalized by MMP9 deletion [76,77]. In MMP9^−/−^, dendritic spine density is reduced and extant spines have a normal morphology [18,47], suggesting that a subset of spiny synapses may be subject to regulation by MMP9. Interestingly, cMD in WTs reduced the spine density to the level observed in MMP9^−/−^ mice, suggesting that visual experience may promote MMP9-sensitive synaptogenesis. Furthermore, in adult mouse V1b, levels of the MMP9 substrate aggrecan are decreased by dark exposure and subsequent light reintroduction, and blocked by the pharmacological inhibition of MMP9 [18,78]. Similarly, the intensity of stained perineuronal nets (PNNs), specialization of the ECM that enshrouds PV+ interneurons, is decreased by brief MD (2 days) during the peak of critical period in WT, but not MMP9^−/−^ mice [47]. PNN density, in turn, regulates the excitatory drive onto PV INs, suggesting that MMP9 may play a role in the regulation of disinhibition in V1b [1]. 

### 3.5. Developmental Expression of MMP9

MMP9 expression peaks at P0 [79,80], nonetheless our findings suggest that MMP9 is dispensable for several forms of activity-dependent synaptic plasticity expressed in perinatal development. Eye-specific segregation of RGC axons, which is driven by spontaneous retinal activity prior to eye opening, was normal in MMP9^−/−^ mice [81,82]. Stimulus selectivity, which is present at eye opening, was grossly normal in adult MMP9^−/−^ mice. Furthermore, cMD induced a significant decrease in the number of thalamocortical afferents on layer 4 pyramidal neuron cell bodies in WTs but not MMP9^−/−^ mice.

The presence of normal contralateral bias of neurons in V1b of MMP9^−/−^ mice demonstrates that the underlying experience-dependent strengthening of contralateral-eye responses is independent of MMP9 activity [2,61], consistent with distinct mechanisms for the development and plasticity of ocular preference [37,62,83,84,85]. Similarly, robust stimulus-selective response potentiation, known to engage mechanisms, distinct from those engaged by MD, also persists in MMP9^−/−^ mice [2,49,86]. The insensitivity of MMP9^−/−^ mice to chronic MD initiated at eye opening suggests that the visual system cannot compensate for the absence of MMP9, and is consistent with the observation that MMP2 activity is not elevated in MMP9^−/−^ mice [18,87].

The contribution of MMP9 to specific forms of synaptic plasticity is predicted to emerge with age, in parallel with the maturation of critical MMP9 substrates. Interestingly, deprived-eye depression in response to MD during the CP persisted in the presence of an MMP9 inhibitor, but the subsequent non-deprived eye strengthening was absent [50]. Non-deprived eye strengthening was also inhibited by manipulations that may reduce MMP9 expression, including deletion of tumor necrosis factor α [88,89]. MMP9 inhibition blocks the enhancement of spatial acuity and contrast sensitivity induced by repetitive engagement of the optomotor reflex in adults [90], although stimulus selective response potentiation is intact in MMP9^−/−^ mice. Together, this suggests that MMP9 is obligatory for the plasticity engaged by cMD from eye opening, but it is dispensable for other forms of plasticity that refine and maintain the mouse visual system.

## 4. Materials and Methods

### 4.1. Subjects

C57BL/6J and MMP9^−/−^ (007084, B6 background) mice were purchased from Jackson Laboratory (Bar Harbor, ME, USA). Equal numbers of adult (>P90, up to P170) males and females were used. The animals were raised in a 12 h light/dark cycle. All procedures conformed to the guidelines of the University of Maryland Institutional Animal Care and Use Committee. Experiments were performed (or subjects were sacrificed) 6 h into the light phase of a 12:12 h light/dark cycle.

### 4.2. Chronic Monocular Deprivation

Chronic monocular deprivation was performed at eye opening (P14). Subjects were anesthetized with 2.5% isoflurane in 100% O_2_. The margins of the upper and lower lids of one eye were trimmed and sutured together using a 5-0 suture kit with polyglycolic acid (CP Medical, Norcross, GA, USA). Subjects were returned to their home cage after recovery at 37 °C for 1–2 h. Subjects were disqualified in the event of suture opening.

### 4.3. Immunohistochemistry

Subjects were anesthetized with 4% isoflurane in O_2_ and perfused with phosphate buffered saline (PBS) followed by 4% paraformaldehyde (PFA) in PBS. The brain was post-fixed in 4% PFA for 24 h, followed by 30% sucrose for 24 h, and cryo-protectant solution for 24 h (0.58 M sucrose, 30% (*v*/*v*) ethylene glycol, 3 mM sodium azide, 0.64 M sodium phosphate, pH 7.4). Coronal sections (40 μm) were cut on a Leica freezing microtome (Model SM 2000R). Sections were blocked with 4% normal goat serum (NGS) containing 0.4% TritonX-100 and 0.1% Tween-20 in 1X PBS for 1 h. The primary antibody mouse anti-vesicular glutamate transporter 2 (VGluT2, RRID: AB_2187552, Millipore, Danvers, MA, USA, 1:500 dilution) was presented in a blocking solution for 18 h, followed by the secondary antibody goat anti-mouse IgG Alexa-488 conjugated (RRID: AB_2534089, Life Technologies, Frederick, MD, USA, 1:1000 dilution). Images were acquired on a Zeiss LSM 710 confocal microscope with a 40× lens (Zeiss, San Diego, CA, USA, Plan-neofluar 40×/1.3 Oil DIC, NA = 1.3). VGluT2 puncta were analyzed using a single Z-section image after the threshold function (auto threshold + 20) was applied, and were then identified by size exclusion (0.15–2.0 μm^2^) using the “analyze particles” function in Fiji (NIH).

### 4.4. Golgi staining and Dendritic Spine Density Analysis

Golgi staining was performed with FD Rapid GolgiStain Kit (FD Neuro Technologies, Columbia, MD, USA), as per the manufacturer’s instructions. The brains were immersed in solution A + B for 7 days at room temperature, and then transferred to solution C for 3 days at 4 °C. Coronal sections (100 μm) were made with a Leica VT100S vibrating microtome and mounted on gelatin-coated slides (FD Neuro Technologies). Neurolucida (MBF Bioscience, St. Albans, VT) with an Olympus BX61 light microscope was used to trace the morphologies of the dendrites and spines. Using a 40× lens (Olympus, Center Valley, PA, USA, Plan N, NA = 0.65), the arbors of basolateral dendrites of layer 4 neurons were traced, followed by sholl analysis in a Neuroexplorer (MBF Bioscience, Willston, VT, USA) to identify the region 75 to 100 μm from the soma, which were then traced using a 100× lens (Olympus Plan N Oil, NA = 1.25). Dendritic protrusions with diameters ≥ 50% than the dendritic shaft diameter were classified as spines, and counted in Neuroexplorer.

### 4.5. Intraocular Injections of Anterograde Tracer

Subjects were anesthetized with 2.5% isoflurane in 100% O_2_. Fluorescently-labeled cholera toxin subunit B solutions (CTB-Alexa 488, or CTB-Alexa 555, Invitrogen, 1 mg/mL dissolved in distilled water, 4 μL) were injected into the temporal region of each eye with a Hamilton syringe [91]. Subjects were perfused 48 h after the injection with phosphate buffered saline (PBS) followed by 4% paraformaldehyde (PFA) in PBS. The brains were post-fixed with 4% PFA for 24 h followed by 30% sucrose for 24 h, and cryo-protectant solution (0.58 M sucrose, 30% (*v*/*v*) ethylene glycol, 3 mM sodium azide, 0.64 M sodium phosphate, pH 7.4) for 24 h prior to sectioning. Coronal sections (40 μm) were made on a Leica (Allendale, NJ, USA) freezing microtome (Model SM 2000R), and then stained with a nuclear marker, DAPI (Sigma, St. Louis, MI, USA).

Confocal images were acquired on a Zeiss LSM 710 confocal microscope with a 10× lens (Zeiss Plan-neofluar 10×/0.30, NA = 0.30). Maximal intensity projections (MIP) of z-stack images (three images with 10 μm interval, at a resolution of 512 × 512 pixel representing 772.9 μm × 772.9 μm) were used for the analysis. Three successive sections through the middle of dLGN were used to quantify the areas of contra- and ipsi-lateral projection defined by fluorescence thresholding (autothreshold + 20) in Fiji. dLGN occupancy was calculated as the ratio of each projection to the total dLGN area.

### 4.6. Virus Injection and Cranial Window Implantation

For two-photon calcium imaging, GCaMP6s was expressed in V1b (AP: 1.0 mm, MD: −3.0 mm, DV: 0.3 mm, contralateral to cMD eye for cMD subjects) using adeno-associated virus (AAV) purchased from Addgene (Watertown, MA, USA, AAV1.hSyn1.mRuby2.GSG.P2A.GCaMP6s.WPRE.SV40, titer: 1.3 × 10^13^ U/mL) injected via a Hamilton syringe attached to a Microsyringe Pump Controller (World Precision Instruments) at a rate of 100 nl/min with a total volume of 30 nl.

A cranial window consisting of three pieces of coverslips (two 3 mm diameter coverslips glued with optical adhesive (Norland71, Edmund Optics, Barrington, NJ, USA) to a 5 mm diameter coverslip) was implanted following a method described by Goldey et al. [92]. The gap between the skull and glass was sealed with a silicone elastomer (Kwik-Sil). Instant adhesive Loctite 454 (Henkel, Richmond, VA, USA) was used to adhere an aluminum head post to the skull, and to cover the exposed skull, and then black dental cement (iron oxide powder, AlphaChemical, Stoughton, MA, USA, mixed with white powder, Dentsply) was used to coat the surface to minimize light reflections. Subjects were imaged after more than at least 3 weeks of recovery.

### 4.7. Two-Photon Imaging

Awake subjects were placed in a holding tube and immobilized by a head post clamp. Prior to the first imaging session, the subjects were placed in the holding tube at least twice for >30 min for habituation. A shield was placed around the gap between the cranial window and the objective lens to block light from the visual stimuli during imaging. A two-photon microscope (ThorLabs, Sterling, VA, USA) controlled by ThorImageLS software with a 16× NA 0.8 water immersion objective lens (Nikon, Melville, NY, USA) was used to acquire the time lapse fluorescence images. A Chameleon Vision Ti: Sapphire laser (Coherent, Santa Clara, CA, USA) was tuned to 940 nm for excitation, and the emitted photons were directed through a 525/50 nm bandpass filter onto a GaAsP photomultiplier tube. The field of view was 370 × 370 μm (512 × 512 pixels) at 150 to 250 μm from the brain surface. The images were acquired at 30 Hz by bidirectional scanning. Before measuring responses to the visual stimuli, the pattern of the GCaMP signal was evaluated to confirm the absence of toxic expression effects [36]. The neurons expressing GCaMP showed a high cytosolic signal with nucleus exclusion (Figure 3A). Time after viral injection or the age of subject did not impact ΔF/F, CBI or % of responsive neurons (Figure 3B, see below for method). If the mean ΔF value during visual stimulation at any direction exceeded 3 × STD of baseline (F_0_) for 40% or more of trials during either contra or ipsi eye stimulation, the neuron was defined as visually responsive. If this criterion was met in response to stimulation of both eyes, the neuron was designated binocular. Using this criterion, the majority of visually-responsive neurons were binocular in both genotypes. Raising the threshold for the definition of visually responsive did not change the % of neurons defined as binocular (Figure 1D and Figure 2D).

For ocular dominance measurements, subjects received monocular visual stimuli controlled by the PsychToolBox plugin in MATLAB, (random order of five repeats of 12 orientations of square grating drifting at 1 Hz) at 0.05 cycle/degree (cpd), 100% contrast (28 cd/m^2^) for 2.5 s interleaved with 2.5 s intensity-matched grey scale) delivered by a 23” display (Acer LCD Monitor, San Jose, CA, USA) 28 cm in front of the eyes. To measure the strength of the visual responses across contrasts and spatial frequencies, visual stimuli were delivered at a range of contrasts (20, 40, 60, 80, and 100%) at 0.05 cycle/degree or a range of spatial frequencies (0.05, 0.10, 0.20, 0.30 and 0.40 cpd) at 100% contrast to the eye contralateral to the cranial imaging window. Movement artifacts were corrected using the Suite2P package [93], using the average intensity of the full image stack as a template. Ring-like regions of interest (ROIs) in the soma area were selected using the averaged image after motion correction, and neuropil subtraction (correction factor, 0.8) was performed to calculate the response amplitude, ΔF/F [31]. To calculate ΔF/F = (F − F_0_)/F_0_, where F corresponds to the fluorescence intensity at a given time point and F_0_ corresponds to mean fluorescent intensity during 1 s before the visual stimulus onset.

### 4.8. Ocular Dominance Analysis

If a mean ΔF value during visual stimulation at any direction exceeded 3 × STD of baseline (F_0_) for 40% or more of trials during either contra or ipsi eye stimulation, the neuron was defined as visually responsive. An ocular dominance score (ODS) of visually responsive neurons was defined as (C − I)/(C + I), where C and I represent the ΔF/F values at a preferred direction delivered to the contra and ipsi eye, respectively. ODSs were binned into five categories: 1 = 1.00–0.60, 2 = 0.59–0.20, 3 = 0.19–−0.19, 4 = −0.2–−0.59, and 5 = −0.60–−1.00. Contralateral bias index (CBI) was calculated as {(N1−N5) + (N2−N4) + Ntot}/2Ntot, where Ntot is the total number of neurons and N1-5 are the numbers of neurons in bins 1 through 5, respectively.

### 4.9. Orientation and Direction Selectivity Analysis

All visually responsive neurons were analyzed. Responses were acquired to visual stimulation (random order of five repeats of 12 different orientations of drifting square grating at 1 Hz at 100% contrast (28 cd/m^2^) for 2.5 s with 2.5 s intensity-matched grey scale) to the eye contralateral to the imaging window at a range of spatial frequencies (0.05, 0.10, 0.20, 0.30, and 0.40 cpd). Orientation selectivity and direction selectivity were calculated at the neuron’s peak spatial frequency. The direction vector, dirR, was defined as dirR = (ΣR(θ) * cosθ/ΣR(θ), ΣR(θ) * sinθ/ΣR(θ)), where R(θ) represents the response (ΔF/F) at the stimulation direction, θ. Direction selectivity was calculated using the direction circular variance, dirCV = 1 − |dirR|, in order to detect small differences (Mazurek et al., 2014). The orientation vector, R was defined as R = (ΣR(θ) * cos2θ/ΣR(θ), ΣR(θ) * sin2θ/ΣR(θ)). The orientation selectivity was calculated using the orientation circular variance, CV = 1 − |R|, in order to detect small differences [94].

### 4.10. Chronic In Vivo Recordings for Stimulus-Selective Response Potentiation (SRP)

Adult mice were anesthetized with 2.5% isoflurane in 100% O_2_ and a handmade 1.2 mm 16-channel shank electrode was implanted in V1b (stereotaxic coordinates from Bregma: anterior/posterior, 2.8 mm; medial/lateral, 3.0 mm; dorsal/ventral, 1.2 mm). After recovery of the righting reflex, animals were administered buprenorphine (0.1 mg/kg, i.p.) for post-surgical analgesia and returned to their home cage. Awake, head fixed recordings began more than 7 days after implantation. Activity was evoked through passive viewing of 200 × 1 s trials of square-wave gratings (0.05 cpd, 100% contrast, reversing at 1 Hz), via MATLAB with Psychtoolbox extensions [95,96], and a grey screen of equal luminance (26 cd/m^2^) for spontaneous responses. The initial visual stimulation was presented for 200 s at a single orientation, then 24 h later, the familiar and novel stimuli were presented for 200 s, respectively [32]. 

### 4.11. Statistical Analyses

An unpaired two-tailed Student’s *t*-test and Mann−Whitney test were used to determine the significance between two independent experimental groups, and a paired Student’s *t*-test was used for two measurements within the same subjects. A one-way ANOVA was used to determine significance between three or more independent experimental groups, as well as repeated measures ANOVA for more than two measures within the same subjects, followed by a Tukey−Kramer post hoc when ANOVA was *p* < 0.05 (JASP). A Kolmogorov−Smirnov (KS) test was used to determine the significance between the distribution of two independent data sets.

## 5. Conclusions

The aim of this study was to identify the contributions of the matrix metalloproteinase MMP9 to postnatal experience-dependent plasticity in the murine visual system. Chronic monocular deprivation initiated at eye opening significantly decreased the strength of the deprived-eye visual responses to all stimulus contrasts and spatial frequencies. Constitutive deletion of MMP9 did not impact the stimulus selectivity of V1b neurons, including ocular preference, as well as tuning for spatial frequency, orientation, and direction. MMP9^−/−^ mice were also completely insensitive to plasticity engaged by cMD. Other forms of experience-dependent plasticity, including stimulus selective response potentiation, were normal in MMP9^−/−^ mice. Thus, the MMP9 activity was dispensable for many aspects of maturation of the visual system, but was obligatory for the plasticity engaged by cMD.

## Data Availability

Data available on request.

## Supplementary Materials

The following supporting information can be downloaded at: https://www.mdpi.com/article/10.3390/ijms23052438/s1.
