# Supplementary material for: Chronic Monocular Deprivation Reveals MMP9-Dependent and -Independent Aspects of Murine Visual System Plasticity
Source: Int J Mol Sci. 2022 Feb 23;23(5):2438. doi: 10.3390/ijms23052438 (PMC8909986; doi:10.3390/ijms23052438)
Supplement: Supplementary file 1 [file ijms-23-02438-s001.zip › ijms-1561002-supplementary.pdf]

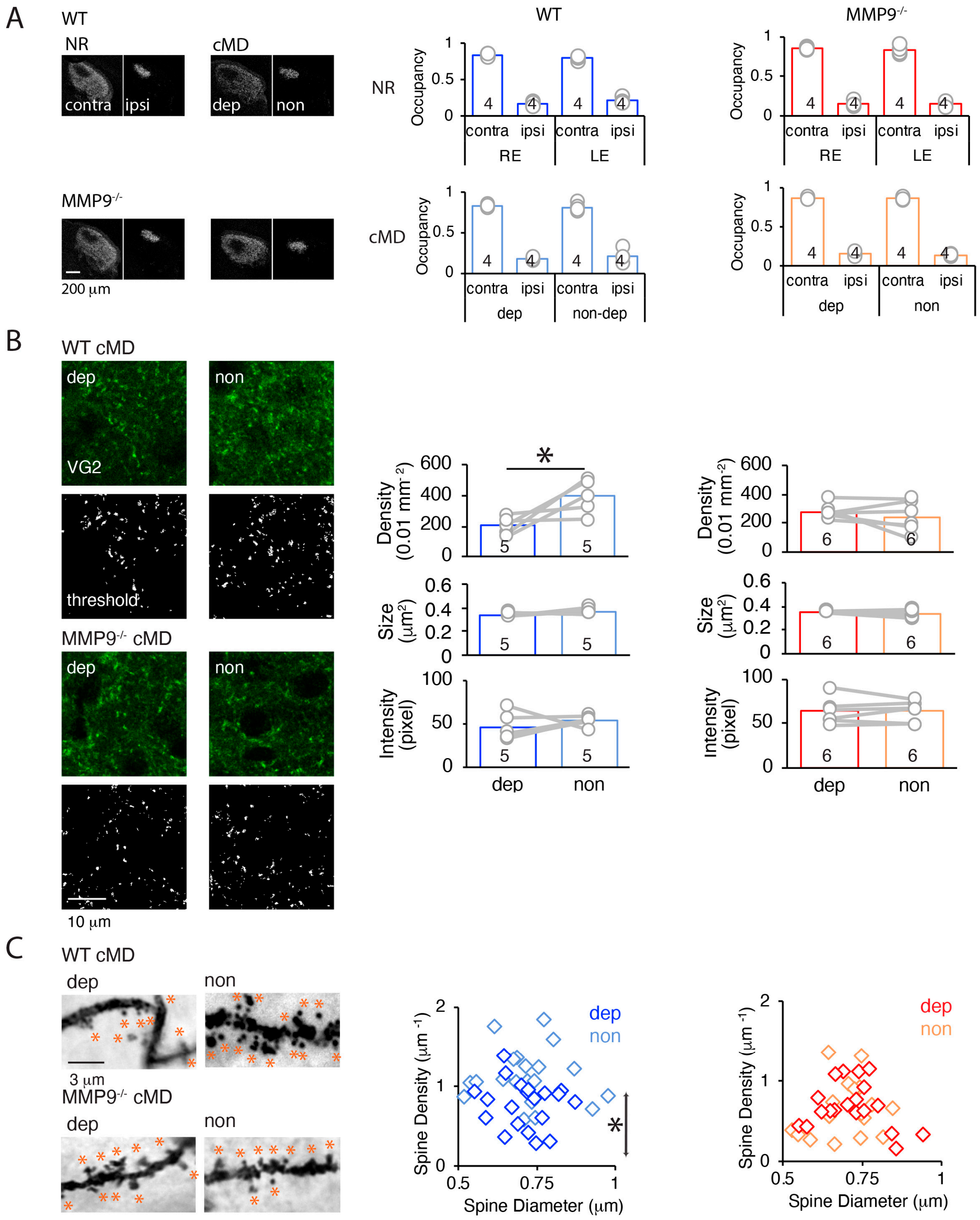

Supplemental Figure S1. Normal eye-specific segregation of retinogeniculate innervation but absence of structural plasticity in V1b layer 4 induced by cMD in MMP9$^{-/-}$ mouse. (**A**) Left: Representative examples of RGC projections to dLGN in NR WT and MMP9$^{-/-}$ mice. Right: Quantification of dLGN occupancy of contra- and ipsi- lateral RGC projections. No difference in WT Normal-reared (NR), right (RE) and left eyes (LE) occupancy in NR following cMD deprived (dep) and non-deprived (non) eyes of cMD. No difference in MMP9$^{-/-}$ NR, right (RE) and left eye (LE) occupancy in NR and cMD. n = 4 subjects. (**B**) Left top: Representative images of VGluT2 (VG2) fluorescence in V1b layer 4 neurons contralateral (dep) and ipsilateral (non) to cMD eye in WT and MMP9$^{-/-}$ mice, and thresholded images used for analysis (bottom). Right: Quantification of VG2 puncta. Significantly lower puncta density in contralateral (dep) relative to ipsilateral (non) to cMD eye in WT. Comparison of dep (dark blue) vs non (light blue) in WT and dep (red) vs non (orange) in MMP9$^{-/-}$ mice. Paired T-tests dep vs non: WT density *p=0.048; WT size: p=0.15; WT intensity: p=0.51; MMP9$^{-/-}$ density: p=0.37; MMP9$^{-/-}$

size: p=0.26; MMP9$^{-/-}$ intensity: p=0.96; n = 5 subjects. (**C**) Left: Representative images of Golgi-stained basolateral dendrites of V1b layer 4 neurons contralateral (dep) and ipsilateral (non) to cMD eye in WT and MMP9$^{-/-}$ mice. Asterisks denote dendritic spines. Right: Quantification of dendritic spine densities (abscissa) and diameters (ordinate) of basolateral dendrites of layer 4 pyramidal neurons measured 75 – 100 µm from soma. Cumulative distributions of layer 4 dendritic spine diameter and density. Comparison of dep (dark blue) vs non (light blue) in WT and dep (red) vs non (orange) in MMP9$^{-/-}$mice. KS tests dep vs non: WT diameter p=0.99; WT density: p=7.25 x 10^{-4}; MMP9$^{-/-}$ diameter: p=0.80; MMP9$^{-/-}$ density: p=0.62; n (subjects, neurons) = (5, 20) for WT, (4, 18) for MMP9$^{-/-}$.
